# Estimating the risk of zoonotic transmission of swine influenza A variant during agricultural fairs in the United States: a mathematical modeling

**DOI:** 10.3389/fvets.2025.1523981

**Published:** 2025-04-01

**Authors:** Dana C. Pittman Ratterree, Sapna Chitlapilly Dass, Martial L. Ndeffo-Mbah

**Affiliations:** ^1^Department of Veterinary Integrative Biosciences, College of Veterinary Medicine and Biomedical Sciences, Texas A&M University, College Station, TX, United States; ^2^Department of Animal Science, College of Agriculture and Life Sciences, Texas A&M University, College Station, TX, United States; ^3^Department of Epidemiology and Biostatistics, School of Public Health, Texas A&M University, College Station, TX, United States

**Keywords:** agricultural fair, swine influenza, mathematical modeling, transmission risk, zoonotic events

## Abstract

**Introduction:**

Agricultural fairs offer a unique interface between humans and swine. We investigate the transmissibility of influenza A variant from pigs to humans using epidemiological data from a 2011 zoonotic outbreak of an influenza H3N2 variant during an agricultural county fair in Pennsylvania.

**Methods:**

We developed a mathematical model for the transmission of a swine influenza pathogen among pigs and humans at an agricultural fair. We fitted our model to the outbreak data to estimate zoonotic transmissibility. We considered nine data-driven scenarios of swine-to-swine basic reproductive number (R_0_) and the number of infected pigs at the start of the fair, and we simulated the zoonotic outbreak dynamics.

**Results:**

We estimated the probability of swine-to-human H3N2v transmission per minute of swine contact for which our model best fitted the data. The probability of transmission of H3N2v per minute of contact with swine among club members was estimated to vary from 0.029 (95% confidence interval (CI): 0.028–0.030), when R_0_ = 2 with 1 initially infected pig, to 0.00099 (0.00095–0.00102), when R_0_ = 6 with 5 initially infected pigs. For attendees, we showed that the probability equals 0.0168 (95% CI: 0.0167–0.0169), when R_0_ = 2 with 1 initially infected pig, and 0.00371 (95% CI: 0.00368–0.00373), when R_0_ = 2 with 5 initially infected pigs. For all scenarios, we estimated H3N2v infection prevalence among club members and attendees to average 12 and 0.7%, respectively.

**Discussion:**

These results show that the transmission risk may vary substantially between club members and attendees and with the underlying disease transmission among pigs. Although fair attendees may have a small transmissibility risk, annual fair attendees represent a large population likely to experience zoonotic events and facilitate the emergence of a potential pandemic influenza variant.

## 1 Introduction

Swine influenza A is a common respiratory disease in domestic pigs (*Sus scrofa domesticus*), clinical signs of the disease include fever, lethargy, coughing, nasal discharge, and labored breathing (1–4). There are three major serotypes of swine influenza circulating: H1N1, H1N2, and H3N2 (4–6). Swine are an important reservoir for zoonotic influenza for humans, referred to as variant influenza, as all three serotypes can cause human disease. The primary risk factor for contracting the swine influenza variant is contact with pigs, as sustained human-to-human transmission is rarely observed (7–11). The Centers for Disease Control (CDC) has documented 546 confirmed zoonotic cases of variant influenza A of swine origin in the United States since 2010 (12, 13). The highest number of cases, 321, were reported during the 2011–2012 flu season; with more than 90% of cases associated with pig exposure at an agricultural fair. The increased number of zoonotic swine influenza cases related to agricultural fairs pose a serious risk for the emergence of a new swine influenza pandemic virus. To mitigate this risk, a pandemic preparedness plan should emphasize the need for a One Health approach to reduce intra- and bidirectional inter-species transmission of influenza viruses at swine exhibitions and beyond (14). This should not only encompass increased monitoring and surveillance activities at agricultural fairs but also the large-scale implementation of effective preventive measures to reduce the risk of zoonotic outbreaks during fairs. Influenza A spillover from swine has significant public health implications due to its observed pandemic potential (11, 15).

Agricultural county fairs offer a unique interface between the general public and livestock such as pigs. In the United States, more than 3,000 county fairs happen each summer and 50 state fairs occur each year (11). These events enable agricultural club members from across a state to bring their hogs to compete for prizes and sell their animals (11). Hundreds of thousands attend these events annually, facilitating direct human-pig contact (11). Influenza A in swine is difficult to detect as many cases are subclinical yet are able to transmit the virus as well as animals with clinical symptoms (7, 16–18). Agricultural county fairs have been the main source of zoonotic swine influenza cases in the United States (11). Roughly half of these zoonotic cases are observed in Ohio and Indiana, but 20 other states throughout the country have reported confirmed variant cases (11, 12). Fairs in Ohio, Michigan, Maryland, and Pennsylvania have documented spillover events linked to agricultural fairs (7, 9, 19–23).

Epidemic models are tools built with a mathematical framework to predict disease spread, assess disease spread, and guide public health response (24, 25). These models are used to analyze disease dynamics through a structured approach. Epidemic models help estimate transmission risk, evaluate control measures, and identify high-risk populations, playing a crucial role in mitigating infectious disease impacts. Applications for epidemic models have been used to analyze swine influenza A in a broad range of production and farm environments (26). However, only one study has been published examining transmission from swine-to-human in an agricultural environment (26).

In this study, we investigated the transmissibility of variant influenza from pigs to humans using epidemiological data from a 2011 zoonotic outbreak of a swine-origin H3N2 variant during an agricultural county fair in Pennsylvania. We expanded on a previous publication that focused on the transmission risk among agricultural club members, by considering additional realistic disease transmission scenarios among pigs as well as evaluating the transmission risk to fair attendees. Club members and attendee transmission risk were evaluated through model fitting data for each population from the 2011 outbreak. We investigated nine data-driven scenarios by varying the basic reproductive number among pigs and the number of infected pigs at the beginning of the fair.

## 2 Methods

### 2.1 Epidemiological model

To estimate the transmissibility of variant influenza A from swine to humans during an agricultural fair, we developed a mathematical model for the joint transmission of the virus between humans and pigs. Our model was similar to a previous dynamic population model for the transmission of H3N2 influenza A variant during an agricultural fair (7). Pathogen transmission among swine was modeled using a simple SIR (Susceptible-Infected-Recovered) model and a SEIR (Susceptible-Exposed-Infected-Recovered) model for disease dynamics among humans. The model dynamics are described by the following ordinary differential equations:


$$\frac{dS_{h}}{dt}=-C\times P\times S_{h}\times \left(\frac{I_{s}}{N_{s}}\right)$$

$$\frac{dE_{h}}{dt}=C\times P\times S_{h}\times \left(\frac{I_{s}}{N_{s}}\right)-\kappa_{h}\times E_{h}$$

$$\frac{dI_{h}}{dt}=\kappa_{h}\times E_{h}-\sigma_{h}\times I_{h}$$

$$\frac{dR_{h}}{dt}=\sigma_{h}\times I_{h}$$

$$\frac{dS_{s}}{dt}=-\beta \times S_{s}\times \left(\frac{I_{s}}{N_{s}}\right)$$

$$\frac{dI_{s}}{dt}=\beta \times S_{s}\times \left(\frac{I_{s}}{N_{s}}\right)-\sigma_{s}\times I_{s}$$

$$\frac{dR_{s}}{dt}=\sigma_{s}\times I_{s}$$

**Figure 1:**
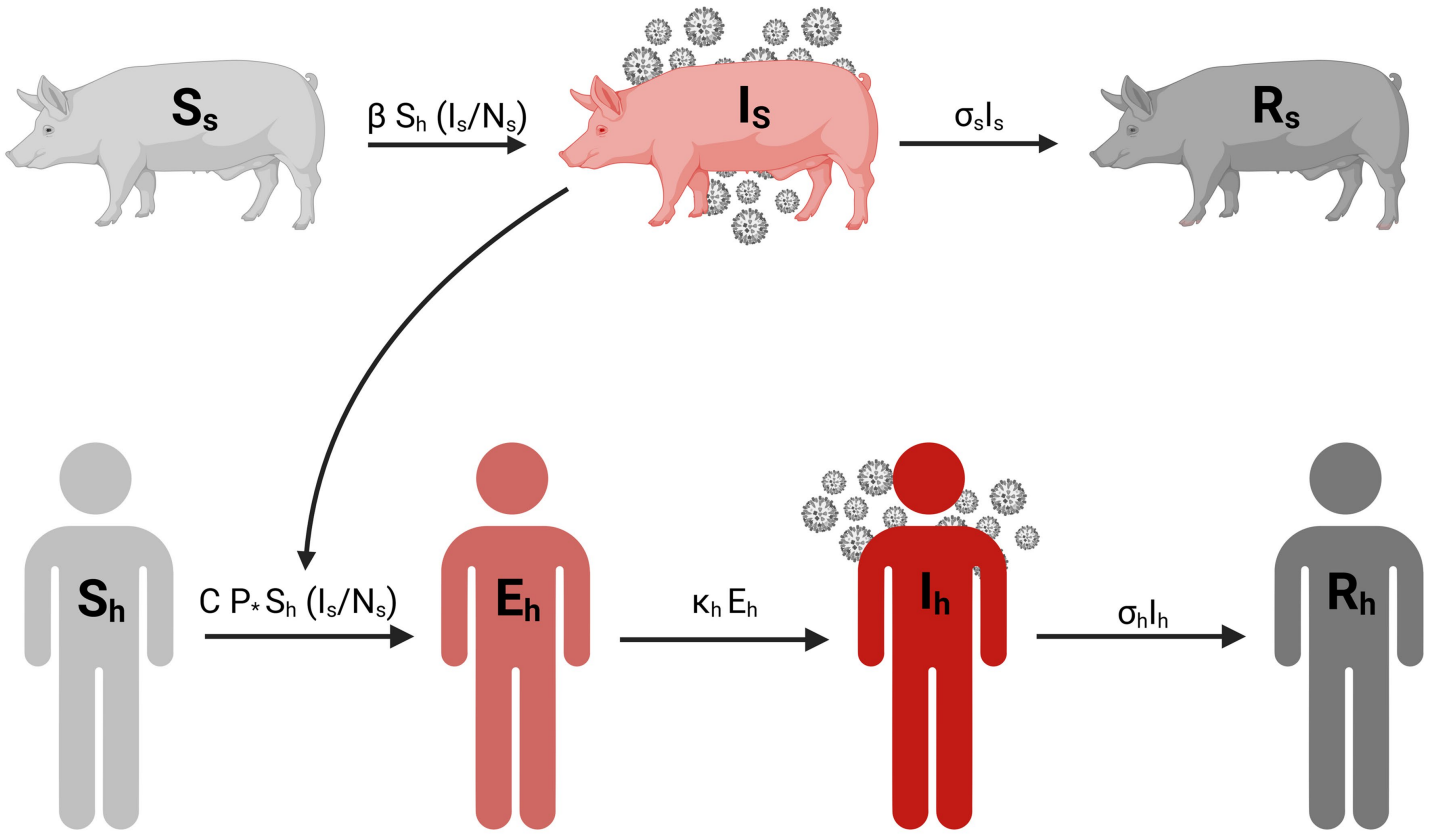
Compartmental diagram for the transmission of influenza A variant between swine and human population. Created in BioRender. Pittman, D. (2025) https://BioRender.com/w17r576.

Where S_h_, E_h_, I_h_, and R_h_ represent susceptible, exposed, infectious, and recovered humans, respectively, and S_s_, I_s_, and R_s_ represent susceptible, infectious, and recovered swine, respectively. *P* represents the probability of transmission per minute of contact with infected swine, *C* is the duration of contact in minutes with infected swine, *N_s_* denotes the total number of swine, 𝜅_h_ is the rate exposed individuals progress to infected (1/incubation), 𝜎_h_ is the human recovery rate is (1/duration of illness), 𝛽 represents swine-to-swine transmission rate of infection, and 𝜎_s_ is the swine recovery rate. The infection rate in humans is proportional to the disease prevalence among swine. Parameter values are provided in Table 1. The descriptive flow diagram of our model is provided in Figure 1.

**Table 1:** Model parameters.

Parameter symbol	Parameter description	Value	Source
–	Total susceptible club member population with swine contact	90	(7)
–	Total susceptible Attendee population with swine contact	14,910 (6,468 age <20 y; 8,442 age ≥ 20 y)	(7, 19)
–	Duration of the fair	9 days	(7, 19)
*C*	Contact duration	60 min (Club member) 5 min (Attendee)	(7)
*P*	Probability of transmission	Model estimated	–
*N_s_*	Total number of exhibited swine	208	(7, 19)
1/κ_h_	Incubation period	2 days	(7, 19)
1/σ_h_	Duration of infection in humans	5 days	(27)
*β*	Swine-to-swine transmission rate	Varied	(3)
1/σ_s_	Duration of infection in swine	5 days	(28, 29)
R_0_	Basic reproduction number for swine	2, 4, 6	(3, 7)

Parameter values for the model came from a combination of literature and empirical observations. During the CDC’s outbreak investigation, a survey was conducted of people who were at the 2011 Pennsylvania fair’s hog exhibition including information on contact duration (7, 19). Both the incubation period and duration of infections in humans estimates were obtained from a systematic review of clinical studies on H3N2v infections in humans (27). Assumptions on those parameters were not tested. We anticipate that the value of these parameters have a negligible impact on our results as there is no human-to-human transmission in the model. For simplicity, we use a fixed rate for the recovery rate in swine (σ_s_) attained from challenge studies comparing vaccinated and unvaccinated pigs (28, 29). Additionally, this assumption by swine viral kinetics (30). The R_0_ values used in this manuscript were obtained from previous estimates of swine influenza outbreaks in swine populations (3).

The model incorporates two modes of transmission: swine-to-swine and swine-to-human (Figure 1). The swine-to-swine transmission rate (*β*) was calculated as the product of the basic reproduction number (R_0_) and the recovery rate for swine (σ_s_). This is a rearrangement of the R_0_ calculation for the simple SIR model (31). Our model assumes new swine infections exclusively through exposure to other infected swine. This assumption is based on the prolonged exposure swine have with each other in the exhibition barn (7, 16–18). Given the duration of human contact with exhibition swine is limited, we assume that new infections in swine are acquired from infected swine rather than humans. Our model assumes no sustained human-to-human transmission of the swine influenza A variant, while contact with infected swine is a well-documented risk factor for zoonotic events (7–9). Furthermore, the epidemiological investigation of this outbreak conducted by the CDC (7, 19) indicated that human-to-swine transmission was unlikely during the fair (7, 19).

### 2.2 Model calibration

We fit our model to data from a 2011 investigation by the Center for Diseases Control and Prevention (CDC) of an H3N2v outbreak in humans who attended the hog exhibition at an agricultural fair in Pennsylvania (7, 19). The duration of the fair was 9 days and 208 pigs were exhibited with over 14,000 general attendees (Table 1) (7, 19). Investigators identified one febrile pig at the start of the fair (7, 19). However, previous studies have reported that around 80% of H3N2 infected pigs are subclinical/asymptomatic (16, 20). So, in our baseline scenario, we assumed that the outbreak among swine was initiated by 5 infected pigs.

Human case data was separated into cases among agricultural club members and general attendees to account for differences in duration and type of contact with the pigs (7, 19). We used a maximum likelihood estimation approach with a normal distribution to fit the deterministic version of our model to the cumulative incidence data for the attendees and club members. With fitting we estimated the value of the probability of transmission for which the model best fit the data. Because the CDC classified the majority of the cases as suspected cases, we conducted a sensitivity analysis like Wong et al. where we assumed 75% of the cases in humans were attributable to H3N2v and refitted the model.

### 2.3 Scenario analysis

We investigated several data-driven scenarios using different swine-to-swine transmission R_0_ values and the number of infected pigs at the start of the fair (3, 16, 18, 32). We considered nine combinations of R_0_ value and the initial number of infected pigs. We set possible values of R_0_ as 2, 4, and 6, derived from previous data-driven studies of influenza A transmission among pigs (3). For the initial number of infected pigs, in addition to our baseline assumption of 5 initial infected pigs described above, we also consider two additional scenarios: (1) as in Wong et al. we assumed that the febrile pig at the start of the Pennsylvania fair was the only initially infected pig, and (2) we assume that there were 3 initially infected pigs under the assumption that 66% of influenza A infected pigs are subclinical (2). These scenarios were used to investigate the impact of the swine-to-swine transmission and the number of infected pigs at the start of the fair on the probability of swine-to-human transmission during a 2011 large-scale zoonotic outbreak of H3N2v during an agricultural county fair in Pennsylvania (19).

To estimate the potential true size of human cases among club members and attendees, we employed a stochastic version of our model. We used the *τ*-leap methodology for stochastic simulation with a Poisson distribution as described by Keeling and Rohani (33). We ran 1,000 simulations for each scenario to capture the range of possible outcomes for prevalence in the pig, club member, and attendee populations. The deterministic and stochastic models were implemented in MATLAB R2023a (version: 9.14.0, Natick, Massachusetts: The MathWorks Inc., 2023). Data visualization and calculations of central tendency for the *τ*-leap simulations were performed in RStudio (version: 2024.9.0.375, Boston, Massachusetts: Posit Software).

## 3 Results

**Figure 2:**
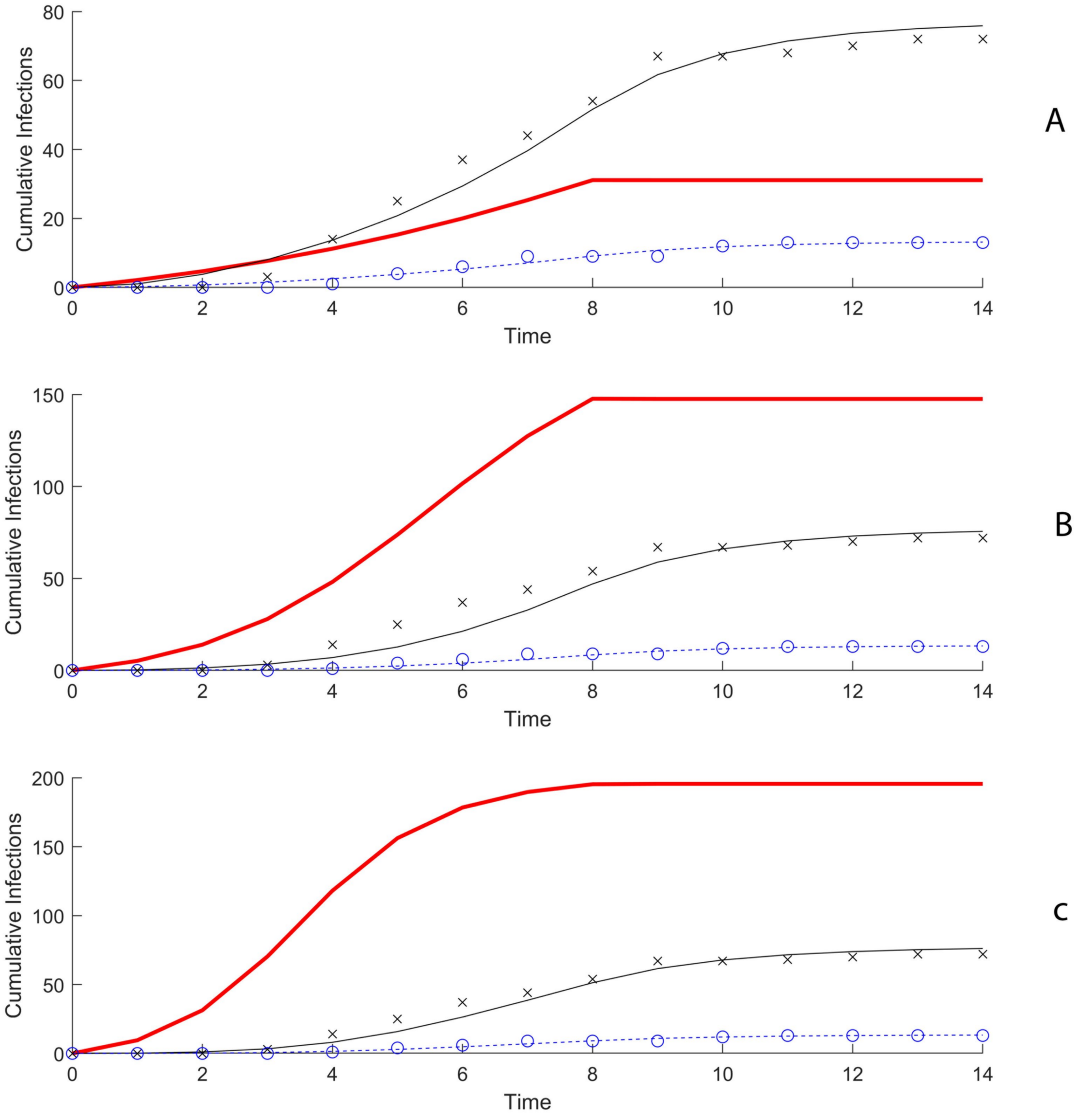
**(A)** Model fitting results using maximum likelihood estimation (MLE) when R_0_ is 2 and 5 initially infected pigs. **(B)** Model fitting results using MLE when R_0_ is 4 and 5 initially infected pigs. **(C)** Model fitting results using MLE when R_0_ is 6 and 5 initially infected pigs.

We considered nine scenarios, each with a different combination of R_0_ values and initial numbers of infected pigs, based on realistic assumptions. For each scenario, our model was fitted to the outbreak data using a Maximum Likelihood Estimation approach (Figure 2). The model fittings enabled us to estimate the probability of swine-to-human transmission of H3N2v for which the model predictions best match the data.

**Figure 3:**
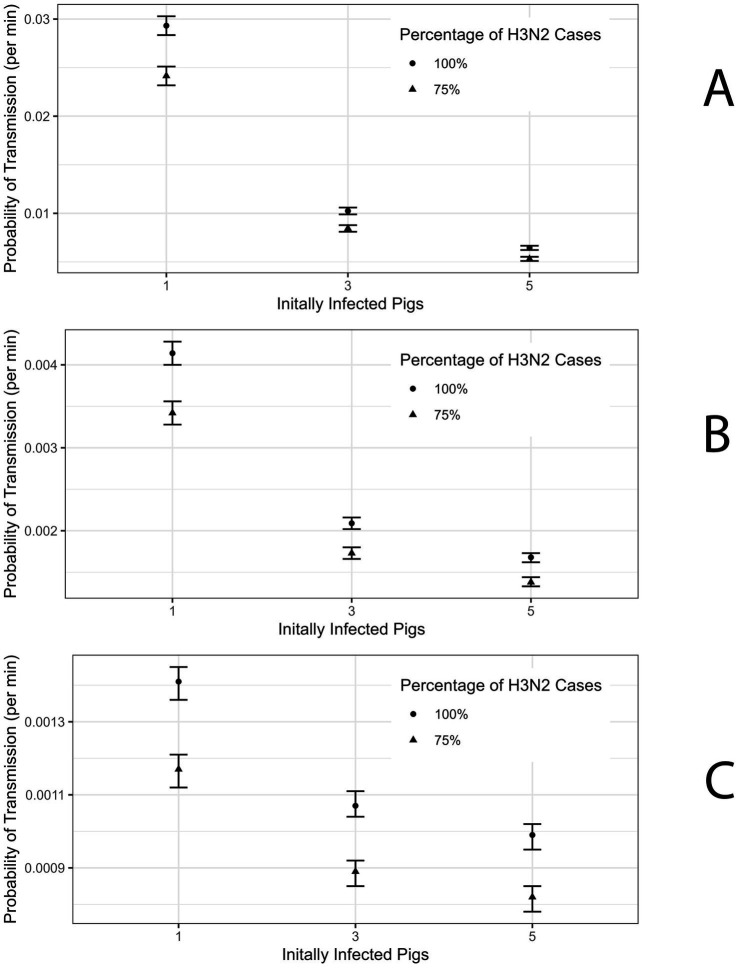
**(A)** Probability of Transmission estimate for R_0_ = 2 for Club member Population with 95% confidence interval (CI). **(B)** Probability of Transmission estimate for R_0_ = 4 for Club member Population with 95% CI. **(C)** Probability of Transmission estimate for R_0_ = 6 for Club member Population with 95% CI.

In the first scenario, R_0_ for swine-to-swine transmission equals 2 and there were 5 initially infected pigs, the probability of transmission of H3N2 to a susceptible club member for each minute of contact with the infectious swine population was estimated to be 0.0062 (95% confidence interval (CI): 0.0064–0.0066) (Figure 3; Supplementary Table 1). When R_0_ is increased to 4 and 6 the probability of transmission for club members was estimated to be 0.00168 (95% CI: 0.00162–0.00173) and 0.00099 (0.00095–0.00102) (Figure 3; Supplementary Table 1). For general attendees, the probability of transmission per minute of contact in the scenario with R_0_ = 2 and 5 initially infected pigs was estimated to be 0.00371 (95% CI: 0.00368–0.00373) (Supplementary Table 2).

In the case of 1 initially infected pig and R_0_ = 2 the probability of transmission for club members is 0.029 (95% CI: 0.028–0.030) (Figure 3; Supplementary Table 1). When R_0_ is 4 and 6 then the probability of transmission for club members was estimated to be 0.0041 (95% CI: 0.0040–0.0042) and 0.0014 (95% CI: 0.00136–0.00145) (Figures 3B,C; Supplementary Table 1). For attendees, the probability of transmission per minute of contact in the scenario with R_0_ = 2 and 1 initially infected pig was estimated at 0.0168 (95% CI: 0.0167–0.0169) (Supplementary Table 2).

In line with Wong et al., we refit the model assuming 75% of the cases among the club members and attendees were attributable to H3N2 (7). The probability of transmission per minute of contact with the infected swine population for club members was estimated to be 0.0053 (95% CI: 0.0051–0.0055) and for attendees was 0.00272 (95% CI: 0.00270–0.00274) under the scenario where R_0_ was 2 and there are 5 initially infected pig (Figure 3A; Supplementary Tables 1, 2). In the scenario where there is 1 initially infected pig the probability of transmission for club members and attendees is 0.0242 (95% CI: 0.0232–0.0251) and 0.01239 (95% CI: 0.01226–0.01251), respectively (Supplementary Tables 1, 2). Estimates of the probability of transmission for club members and attendees for the remaining scenarios are presented in Supplementary Tables 1, 2.

**Figure 4:**
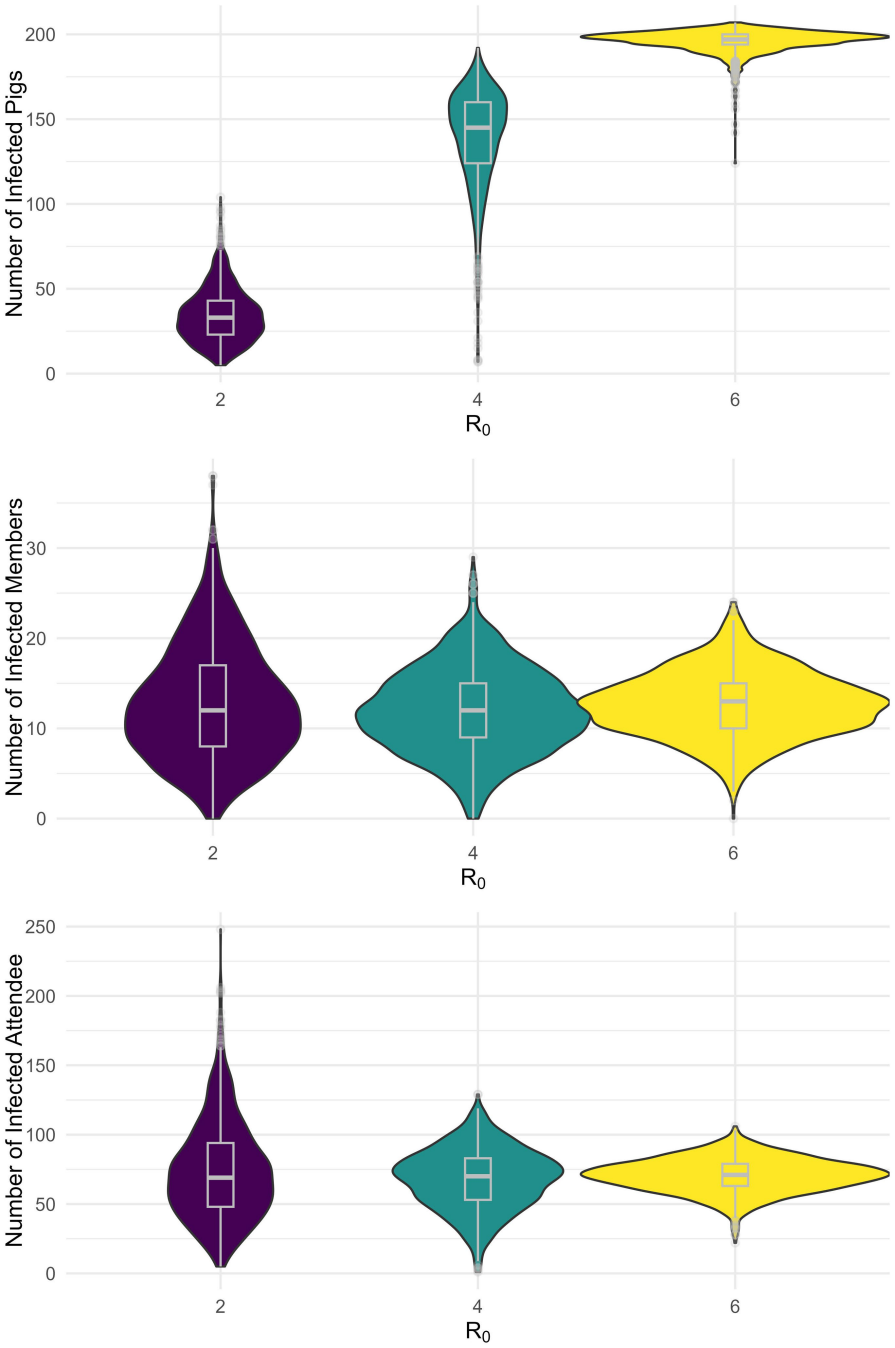
Stochastic simulation of the infection prevalence by population for five initially infected pigs.

Next, we conducted 1,000 stochastic simulations and calculated the expected cumulative infection prevalence among swine, club members, and attendees during the fair for each of the nine scenarios (Supplementary Table 3; Figure 4; Supplementary Figures 1, 2). In the scenario where 5 pigs are initially infected and R_0_ equals 2, 4, and 6 the median prevalence of infected swine was 15.9% (Interquartile Range (IQR): 11.1–20.7%), 69.7% (IQR: 59.6–76.9%), and 94.7% (IQR: 93.3–96.2%), respectively (Figure 4). These correspond to 33 (IQR: 23–43), 145 (IQR: 124–160), and 197 (IQR: 194–200) infected pigs, respectively. Among club members, the prevalence was 13.3% (IQR: 8.9–18.9%), 13.3% (IQR: 10.0–16.7%), and 14.4% (IQR: 11.1–16.7%) when R_0_ was 2, 4, and 6, respectively, with 5 initially infected pig (Figure 4). These correspond to 12 (IQR: 8–17), 12 (IQR: 9–15), and 13 (IQR: 10–15) infected club members, respectively (Supplementary Table 3). For attendees, the prevalence was 0.7% (IQR: 0.5–0.9), 0.7% (IQR: 0.5–0.9), and 0.5% (IQR: 0.6–0.8) across R_0_ values of 2, 4, and 6, respectively (Figure 4). These correspond to 69 (IQR: 48–94), 70 (IQR: 53–83), and 71 (IQR: 63–79) infected attendees, respectively. Similar qualitative results were obtained for the other scenarios (Supplementary Table 3).

Assuming 75% of cases were attributable to H3N2, the median prevalence of infections among club members in the scenario with 5 initially infected pigs and R_0_ was equal to 2, 4, and 6 was 11.1% (IQR: 6.7–15.6), 11.1% (IQR: 7.8–14.4), and 11.1% (IQR: 8.9–14.4), respectively (Supplementary Table 4). These correspond to 10 (IQR: 6–14), 10 (IQR: 7–13), and 10 (IQR: 8–13) infected members, respectively. For attendees, the prevalence when R_0_ was equal to 2, 4, and 6 was 0.5% (IQR: 0.3–0.7), 0.5% (IQR: 0.4–0.6), and 0.5% (IQR: 0.4–0.6), respectively (Supplementary Table 4). These correspond to 51.5 (IQR: 32–71), 50 (IQR: 40–62), and 52 (IQR: 45–58) infected attendees, respectively.

## 4 Discussion

In this study, we estimated the probability of swine-to-human transmission of an influenza A virus variant during agricultural county fairs in the US. We analyzed a previously modeled zoonotic outbreak of swine influenza at a 2011 Pennsylvania agricultural county fair (7, 19). We considered several realistic scenarios of swine-to-swine transmission dynamics under different values of swine-to-swine R_0_ and the number of infected pigs at the start of the fair. This analysis showed that swine prevalence increases with R_0_ values and/or the number of initially infected pigs. As our model assumes that human infections are proportional to the product of swine infection prevalence and human-swine contact rate, we anticipated that the presence of high swine prevalence will require lower per-minute/contact probability, than in the case of low swine prevalence, to generate the same number of infected human cases. In fitting our model to empirical data of human cases, during the 2011 Pennsylvania H3N2v outbreak, under different values of R₀ and the number of initially infected pigs, our estimates for the per-minute transmission probability were shown to decrease with increased R₀ values and the number initially infected pigs (Figure 3; Supplementary Tables 1, 2).

Our results do not suggest that the risk of zoonotic swine influenza infection decreases with higher swine-to-swine R_0_ and the number of initially infected pigs; this was simply a direct effect of fitting all scenarios to the same empirical outbreak data. What should be concluded from these results is the probability of transmission is dependent on disease transmission dynamics among pigs. This was confirmed by our outbreak simulations which predicted a similar average outbreak size among club members, and attendees, across the nine scenarios, respectively (Figure 4). The observed marginal differences in median prevalence and interquartile ranges across the different scenarios simply reflect the intrinsic variability from stochastic simulations.

In the US, H3N2 is most associated with pigs. However, agricultural fairs generally host several livestock species alongside pigs. This unique situation may increase the risk of H3N2 spillover events from pigs to other livestock species. With the recent introduction of the avian influenza H5N1 in cattle, goats, and other mammalian species, fairs can become ideal settings for H5N1 spillover events among various host species and viral reassortments that can present a serious threat for the emergence of novel, highly pathogenic, and highly transmissible influenza virus variant.

The attack rate for influenza A among swine is generally high (3, 18). This conclusion is supported by the high viral loads found in exhibition barns and observed prevalence in swine (18, 34–36). Despite the high attack rate, cases in swine can be difficult to identify as these infections can be subclinical but shed the virus as much as ILI pigs (16). A recent large-scale epidemiological study investigating the spread of influenza A viruses in pig shows (jackpot state and national shows, and county fairs) across eight US states, showed a significant variation in influenza prevalence across show types (37). They estimated that, annually, around 60% of county fairs do not experience an influenza outbreak among show pigs (37). However, they showed that during swine influenza outbreaks in county fairs, more than 75% of pigs were infected on average (37). This observation is consistent with a swine-to-swine R_0_ value between 4 and 6 as observed in our results rather than an R_0_ value equal to 2 as Wong et al. assumed in their model (3, 7).

Though the reported cases of spillover events during agricultural fairs are generally low [cumulative less than 500 cases since 2011 (37)], this should not be interpreted as a low or marginal risk of infection (7). Zoonotic influenza case finding is difficult because most cases are subclinical or present mild influenza-like illness (ILI) symptoms and those who do not have severe manifestations may not seek medical attention. The limited number of people seeking care for ILI after swine exposure makes the reported attack rate artificially low, creating the perception of a low zoonotic infection risk. Additionally, people who do seek out outpatient treatment may not be tested for the swine influenza variant unless they mention the onset of symptoms after visiting an animal exhibition (10).

A literature review of mechanistic models of swine influenza identified Wong et al. as the only existing model on the transmission of swine influenza in the context of agricultural fairs (26). Furthermore, Wong et al.’s epidemiological investigation and subsequent estimation of the probability of swine-to-human transmission of an influenza A variant has been applied to modeling influenza A spillover events for farmworkers (7, 38, 39). This study extended Wong et al. by considering several realistic scenarios of swine-to-swine transmission of H3N2 at the 2011 Pennsylvania fair outbreak that were not accounted for. Wong et al. assumed an R₀ value of 2 for swine-to-swine transmission under the condition that only one pig was infected at the start of the fair. Under similar conditions, our estimates for the probability of swine-to-human transmission among club members were shown to be equal to Wong et al. estimates. This scenario resulted in low infection prevalence among show pigs which is inconsistent with empirical observations on swine influenza outbreaks at agricultural fairs (37). By considering higher R₀ values and numbers of initially infected pigs, which are consistent with empirical observations (34, 37), we obtained lower estimates of the probability of transmission per minute of contact.

Wong et al. used their probability of transmission calculated from the retrospective epidemiological investigation of club members to calculate estimates for the number of infected general attendees, this assumption assumed the type of contact agricultural club members and general attendees have is the same (7). In addition, this study accounted for differential transmission risk between swine exhibitors (club members) and general fair attendees, as both groups have different exposure and types of interactions with show pigs. As expected, club members were shown to experience a higher probability of infection per minute than general fair attendees. Altogether, this study provides a broader and more in-depth analysis of the risk of swine-to-human transmission of swine influenza variant during agricultural fairs.

Like any mathematical model, ours has several limitations driven by model assumptions and the quality of the data used to inform the model’s design and parameterization. For example, influenza A infection among pigs has been shown to be mostly subclinical with clinically and subclinically infected pigs may contribute differentially to swine-to-swine and swine-to-human transmission (16). However, for the sake of simplicity, our model did not explicitly distinguish clinically and subclinically infected pigs and also did not account for the potential impact of the latent infection period on swine-to-swine disease dynamics. Moreover, our model was fitted to a single outbreak dataset to estimate the swine-to-human transmission probability of the influenza A variant. For simplicity, we do not consider alternative formulations of the probability of transmission. This was due to limited available good-quality data on zoonotic influenza outbreaks in agricultural fairs.

The few epidemiological investigations that have reported on these outbreaks generally do not distinguish cases among general attendees and exhibitioners/agricultural club members or provide necessary information about fair activities such as attendance and human-swine contacts which are paramount for model parameterization (22, 23, 40). As exhibitioners experience closer and longer contact with pigs than an average fair visitor, they are at higher risk of contracting the disease than a general fair attendee. However, exhibitors are also more likely to have higher pre-existing immunity levels to circulating swine influenza variants than the average fair visitor as they are more likely to have previously experienced exposure to infected pigs.

The current modeling study is limited to estimating the risk of zoonotic transmission of swine influenza at agricultural fairs. These estimates will be paramount for future modeling studies to design and evaluate effective prevention and mitigation strategies for the control of zoonotic influenza outbreaks at agricultural fairs. Mathematical models of swine influenza transmission at agricultural fairs should be extended to account for these potential risk heterogeneities between exhibitors and general fair attendees which were not considered in our model. Such a model extension may provide a better estimate of the risk of zoonotic events during agricultural fairs. But to accurately parameterize these models, future epidemiological investigations of zoonotic swine influenza outbreaks should provide, at least, detailed information on case incidence and risk activities/contacts among exhibitors and attendees, and prevalence among show pigs.

## 5 Conclusion

In this study, we use computational modeling approaches to investigate the transmission risk of an emerging swine influenza zoonotic pathogen during agricultural fairs. We extend a previous modeling study by Wong et al. (7), that estimated the swine-to-human transmission risk and zoonotic infection burden among all fair attendees during a 2011 H3N2v outbreak at an agricultural county fair in Pennsylvania. The study extension was done by considering several realistic scenarios, for swine-to-swine transmission risk and the number of initially infected pigs, that were not considered in Wong et al. (7). Findings show that the probability of swine-to-human transmission of an influenza zoonotic pathogen varies substantially between agricultural club members and other fair attendees, and these probabilities may vary with disease prevalence among pigs. We estimated that as many as 110 fair-associated H3N2v cases may have occurred during the 2011 Pennsylvania zoonotic outbreak.

Zoonotic swine influenza outbreaks can play a central role in the emergence of a new pandemic influenza virus. Therefore, improving our understanding of zoonotic swine influenza outbreaks and how best to prevent them is paramount for designing more effective pandemic preparedness strategies. Mathematical modeling of zoonotic influenza can be a pivotal tool not only for identifying and quantifying outbreak risk factors in different settings but also for designing and evaluating the effectiveness of surveillance and control measures. Future work should investigate the potential impact of control measures such as swine vaccination, screening and isolation, and shortening the duration of swine exhibition for mitigating the risk and burden of zoonotic outbreaks during agricultural fairs using epidemic modeling methods. To improve the parameterization of these outbreak models, future epidemiological investigations of zoonotic outbreaks at fairs should consider collecting information on fair activities such as attendance and human-swine contact types and frequency. This information is paramount for model parameterization (22, 23, 40). As exhibitioners experience closer and longer contact with pigs than an average fair visitor, they are at higher risk of contracting the disease than a general fair attendee. However, exhibitors are also more likely to have higher pre-existing immunity levels to circulating swine influenza variants than the average fair visitor as they are more likely to have previously experienced exposure to infected pigs. Obtaining such information will greatly increase the accuracy of model predictions.

## Data Availability

The original contributions presented in the study are included in the article/Supplementary material, further inquiries can be directed to the corresponding authors.
