# Supplementary material for: Estimating the risk of zoonotic transmission of swine influenza A variant during agricultural fairs in the United States: a mathematical modeling
Source: Front Vet Sci. 2025 Apr 1;12:1523981. doi: 10.3389/fvets.2025.1523981 (PMC11997979; doi:10.3389/fvets.2025.1523981)
Supplement: Supplementary file 1 [file Supplementary_file_1.docx]

Supplementary Material

# Supplementary Figures and Tables

## Supplementary Tables

**Supplementary Table 1** The probability of transmission for Members

| Initially infected pigs (#) | R_0_ | Portion of cases attributed (%) | Probability of Transmission (Pm) | Confidence Interval (95%) |
| --- | --- | --- | --- | --- |
| 1 | 2 | 100 | 0.02932 | (0.02835, 0.03029) |
| 3 | 2 | 100 | 0.01024 | (0.00989, 0.01059) |
| 5 | 2 | 100 | 0.00644 | (0.00622, 0.00666) |
| 1 | 2 | 75 | 0.02415 | (0.02318, 0.02511) |
| 3 | 2 | 75 | 0.00844 | (0.00810, 0.00878) |
| 5 | 2 | 75 | 0.00530 | (0.00509, 0.00552) |
| 1 | 4 | 100 | 0.00414 | (0.00400, 0.00428) |
| 3 | 4 | 100 | 0.00209 | (0.00202, 0.00216) |
| 5 | 4 | 100 | 0.00168 | (0.00162, 0.00173) |
| 1 | 4 | 75 | 0.00342 | (0.00328, 0.00356) |
| 3 | 4 | 75 | 0.00173 | (0.00166, 0.00180) |
| 5 | 4 | 75 | 0.00138 | (0.00133, 0.00144) |
| 1 | 6 | 100 | 0.00141 | (0.00136, 0.00145) |
| 3 | 6 | 100 | 0.00107 | (0.00104, 0.00111) |
| 5 | 6 | 100 | 0.00099 | (0.00095, 0.00102) |
| 1 | 6 | 75 | 0.00117 | (0.00112, 0.00121) |
| 3 | 6 | 75 | 0.00089 | (0.00085, 0.00092) |
| 5 | 6 | 75 | 0.00082 | (0.00078, 0.00085) |

**Supplementary Table 2** The probability of transmission parameter value for Attendees

| Initially infected pigs | R_0_ | Portion of cases attributed (%) | Probability of Transmission (Pa) | Confidence Interval (95%) |
| --- | --- | --- | --- | --- |
| 1 | 2 | 100 | 0.01683 | (0.01673, 0.01694) |
| 3 | 2 | 100 | 0.00578 | (0.00574, 0.00582) |
| 5 | 2 | 100 | 0.00371 | (0.00368, 0.00373) |
| 1 | 2 | 75 | 0.01239 | (0.01226, 0.01251) |
| 3 | 2 | 75 | 0.00438 | (0.00435, 0.00441) |
| 5 | 2 | 75 | 0.00272 | (0.00270, 0.00274) |
| 1 | 4 | 100 | 0.00232 | (0.00230, 0.00233) |
| 3 | 4 | 100 | 0.00123 | (0.00123, 0.00124) |
| 5 | 4 | 100 | 0.00095 | (0.00094961, 0.00096014) |
| 1 | 4 | 75 | 0.00170 | (0.00169, 0.00171) |
| 3 | 4 | 75 | 0.00090 | (0.00089694, 0.00091052) |
| 5 | 4 | 75 | 0.00070 | (0.00069470, 0.00070521) |
| 1 | 6 | 100 | 0.00080 | (0.00079067, 0.00079948) |
| 3 | 6 | 100 | 0.00061633 | (0.00061294, 0.00061972) |
| 5 | 6 | 100 | 0.00056243 | (0.00055934,  0.00056552) |
| 1 | 6 | 75 | 0.00058 | (0.0005784, 0.00058720) |
| 3 | 6 | 75 | 0.00045 | (0.0004843, 0.00045520) |
| 5 | 6 | 75 | 0.00041 | (0.00040924,  0.00041541) |

**Supplementary Table 3** Summary statistics of stochastic simulations when 100% of suspected H3N2 cases are attributable to fair.

| Population | Number of Infected Pig(s) | R_0_ | Median Prevalence (%) | Q1 Prevalence (25%) | Q3  Prevalence (75%) | Median Case (#) | Q1 Case | Q3 Case |
| --- | --- | --- | --- | --- | --- | --- | --- | --- |
| Pig | 1 | 2 | 1.4 | 0.5 | 5.3 | 3 | 1 | 11 |
| Pig | 1 | 4 | 26.9 | 2.8 | 47.1 | 56 | 5.75 | 98 |
| Pig | 1 | 6 | 83.7 | 60.6 | 90.9 | 174 | 126 | 189 |
| Member | 1 | 2 | 7.8 | 1.1 | 20.0 | 7 | 1 | 18 |
| Member | 1 | 4 | 8.9 | 1.1 | 17.8 | 8 | 1 | 16 |
| Member | 1 | 6 | 11.1 | 5.6 | 15.6 | 10 | 5 | 14 |
| Attendee | 1 | 2 | 0.4 | 0.1 | 1.0 | 42.5 | 8 | 101 |
| Attendee | 1 | 4 | 0.4 | 0.1 | 0.9 | 45 | 6 | 89 |
| Attendee | 1 | 6 | 0.6 | 0.3 | 0.8 | 59 | 31 | 79 |
| Pig | 3 | 2 | 9.1 | 4.8 | 14.4 | 19 | 10 | 30 |
| Pig | 3 | 4 | 57.2 | 42.3 | 69.2 | 119 | 88 | 144 |
| Pig | 3 | 6 | 92.8 | 89.4 | 95.2 | 193 | 186 | 198 |
| Member | 3 | 2 | 12.2 | 6.7 | 18.9 | 11 | 6 | 17 |
| Member | 3 | 4 | 12.2 | 7.8 | 16.7 | 11 | 7 | 15 |
| Member | 3 | 6 | 13.3 | 10 | 15.8 | 12 | 9 | 14.25 |
| Attendee | 3 | 2 | 0.6 | 0.3 | 9.7 | 62.5 | 33 | 97 |
| Attendee | 3 | 4 | 0.6 | 0.4 | 0.9 | 64 | 42 | 87 |
| Attendee | 3 | 6 | 0.7 | 0.6 | 0.8 | 70 | 59 | 79 |
| Pig | 5 | 2 | 15.9 | 11.1 | 20.7 | 33 | 23 | 43 |
| Pig | 5 | 4 | 69.7 | 59.6 | 76.9 | 145 | 124 | 160 |
| Pig | 5 | 6 | 94.7 | 93.3 | 96.2 | 197 | 194 | 200 |
| Member | 5 | 2 | 13.3 | 8.9 | 18.9 | 12 | 8 | 17 |
| Member | 5 | 4 | 13.3 | 10.0 | 16.7 | 12 | 9 | 15 |
| Member | 5 | 6 | 14.4 | 11.1 | 16.7 | 13 | 10 | 15 |
| Attendee | 5 | 2 | 0.7 | 0.5 | 0.9 | 69 | 48 | 94 |
| Attendee | 5 | 4 | 0.7 | 0.5 | 0.8 | 70 | 53 | 83 |
| Attendee | 5 | 6 | 0.7 | 0.6 | 0.8 | 71 | 63 | 79 |

**Supplementary Table 4** Summary statistics of stochastic simulation when 75% of suspected cases are attributable to fair.

| Population | Number of Infected Pig(s) | R_0_ | Median Prevalence (%) | Q1 Prevalence (25%) | Q3 Prevalence(75%) | Median Case (#) | Q1 Case | Q3 Case |
| --- | --- | --- | --- | --- | --- | --- | --- | --- |
| Pig | 1 | 2 | 1.9 | 0.5 | 5.8 | 4 | 1 | 12 |
| Pig | 1 | 4 | 28.6 | 4.8 | 48.6 | 59.5 | 10 | 101 |
| Pig | 1 | 6 | 84.1 | 65.3 | 90.4 | 175 | 135.75 | 188 |
| Member | 1 | 2 | 7.8 | 2.2 | 17.8 | 7 | 2 | 16 |
| Member | 1 | 4 | 8.9 | 1.1 | 15.6 | 8 | 1 | 14 |
| Member | 1 | 6 | 8.9 | 4.4 | 13.3 | 8 | 4 | 12 |
| Attendee | 1 | 2 | 0.4 | 0.1 | 0.8 | 36 | 7 | 83.25 |
| Attendee | 1 | 4 | 0.4 | 0.1 | 0.7 | 35.5 | 7 | 68 |
| Attendee | 1 | 6 | 0.4 | 0.2 | 0.6 | 44 | 23.75 | 58 |
| Pig | 3 | 2 | 9.6 | 5.8 | 14.9 | 20 | 12 | 31 |
| Pig | 3 | 4 | 58.7 | 43.8 | 69.7 | 122 | 91 | 145 |
| Pig | 3 | 6 | 93.3 | 89.4 | 95.2 | 194 | 186 | 198 |
| Member | 3 | 2 | 10.0 | 5.6 | 16.7 | 9 | 5 | 15 |
| Member | 3 | 4 | 10.0 | 6.7 | 14.4 | 9 | 6 | 13 |
| Member | 3 | 6 | 11.1 | 8.9 | 12 | 10 | 8 | 12 |
| Attendee | 3 | 2 | 0.5 | 0.3 | 0.7 | 49 | 29 | 75 |
| Attendee | 3 | 4 | 0.5 | 0.3 | 0.6 | 49 | 32.75 | 64 |
| Attendee | 3 | 6 | 0.5 | 0.4 | 0.5 | 51 | 43 | 58 |
| Pig | 5 | 2 | 15.9 | 10.6 | 22.1 | 33 | 22 | 46 |
| Pig | 5 | 4 | 69.7 | 61.1 | 76.9 | 145 | 127 | 160 |
| Pig | 5 | 6 | 94.7 | 92.8 | 96.2 | 197 | 200 | 200 |
| Member | 5 | 2 | 11.1 | 6.7 | 15.6 | 10 | 6 | 14 |
| Member | 5 | 4 | 11.1 | 7.8 | 14.4 | 10 | 7 | 13 |
| Member | 5 | 6 | 11.1 | 8.9 | 14.4 | 10 | 8 | 13 |
| Attendee | 5 | 2 | 0.5 | 0.3 | 0.7 | 51.5 | 32 | 71 |
| Attendee | 5 | 4 | 0.5 | 0.4 | 0.6 | 50 | 40 | 62 |
| Attendee | 5 | 6 | 0.5 | 0.4 | 0.6 | 52 | 45 | 58 |

## Supplementary Figures


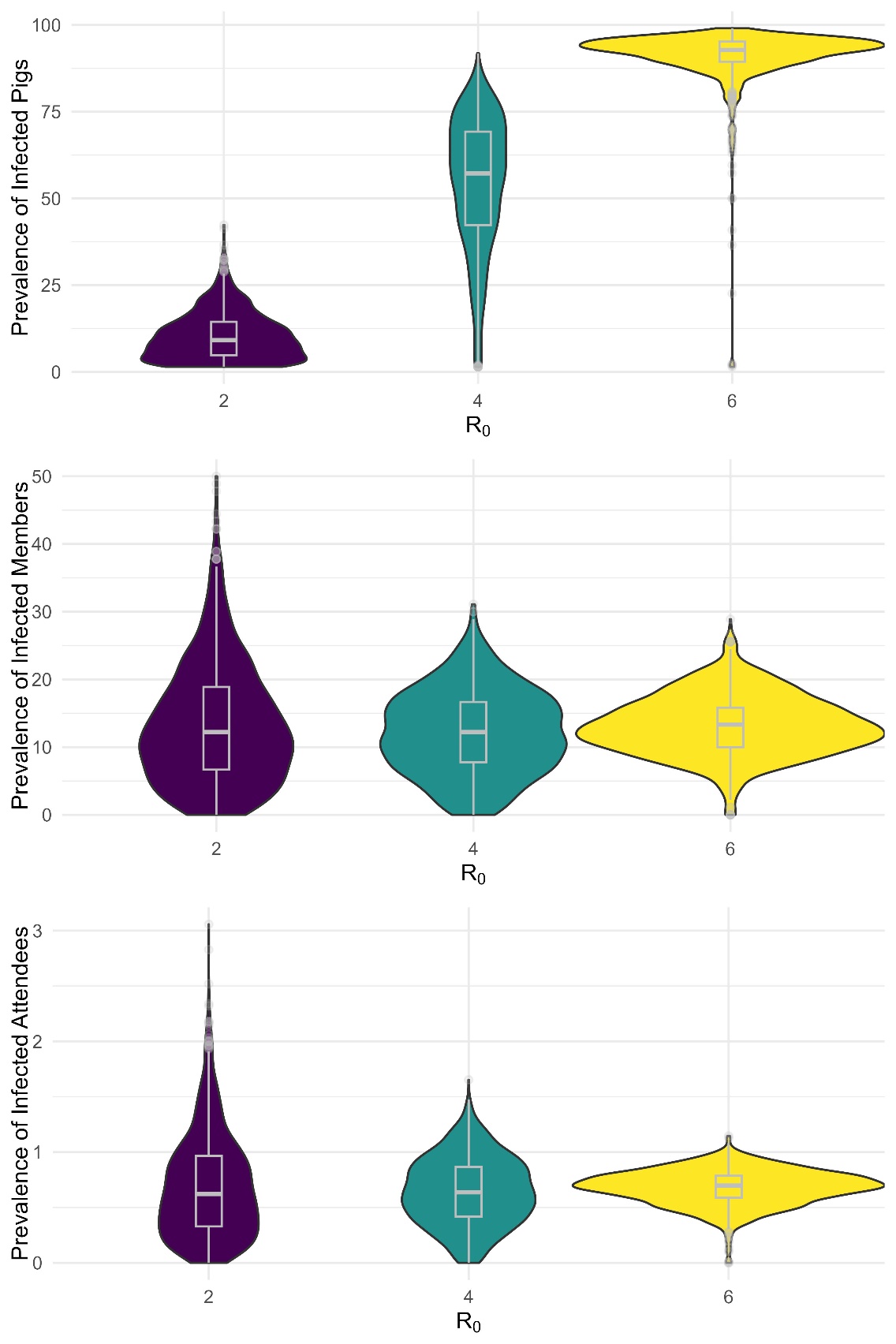


A

B

C

**Supplementary Figure 1.** Stochastic simulation of the infection prevalence by population for three initially infected pigs.

**
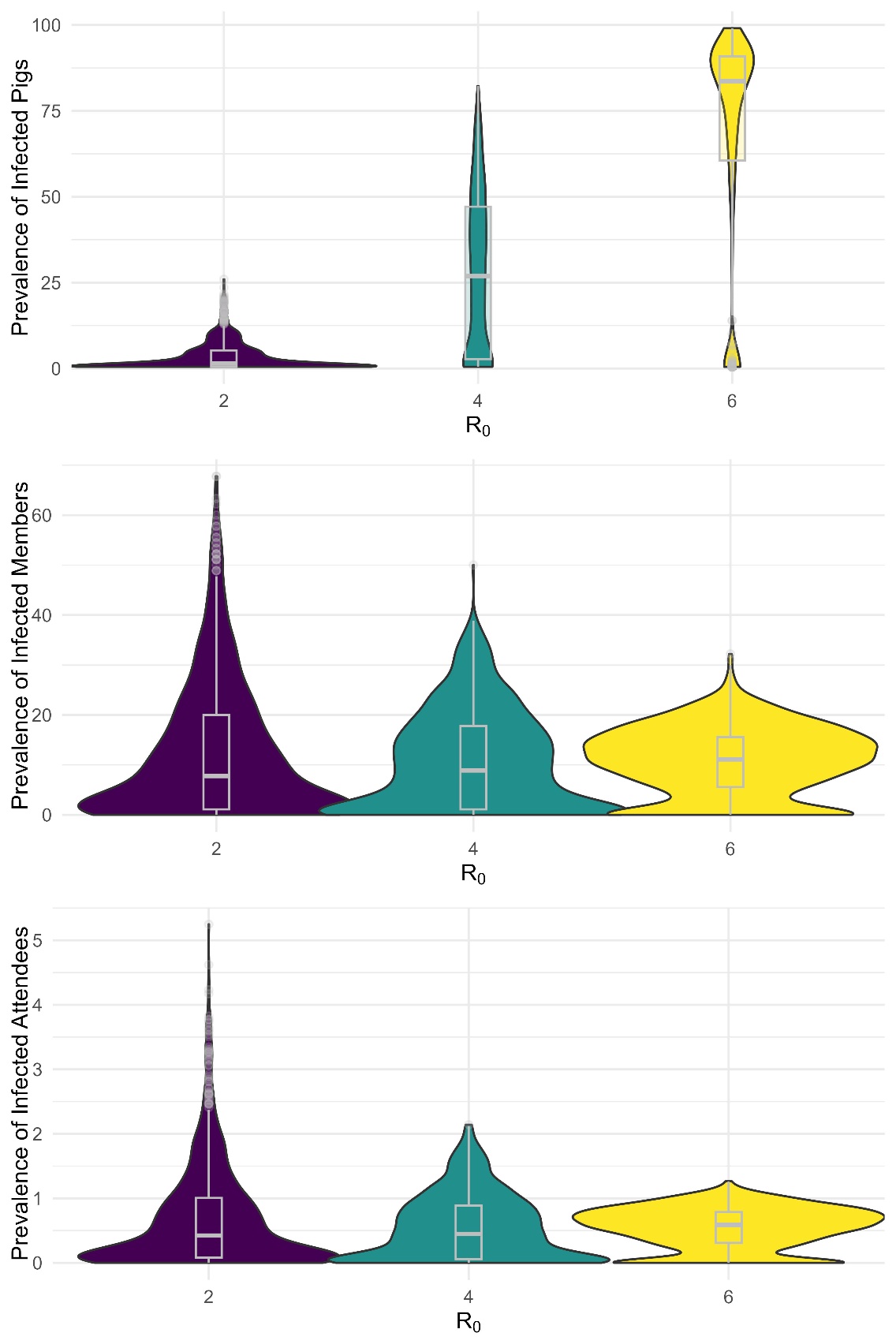
**

C

B

A

**Supplementary Figure 2.** Stochastic simulation of the infection prevalence by population for one initially infected pigs.
